# The Effect of the COVID-19 Pandemic on Riding Lesson Barns and Summer Camps in Ontario

**DOI:** 10.3390/ani10122412

**Published:** 2020-12-17

**Authors:** Katrina Merkies, Caleigh Copelin, Elizabeth Crouchman, Amanda St-Onge

**Affiliations:** Department of Animal Biosciences, University of Guelph, Guelph, ON N1G 2W1, Canada; ccopelin@uoguelph.ca (C.C.); ecrouchm@uoguelph.ca (E.C.); astonge@uoguelph.ca (A.S.-O.)

**Keywords:** facemasks, protocols, hygiene, positives

## Abstract

**Simple Summary:**

Many riding lesson facilities are experiencing financial hardship due to COVID-19 pandemic restrictions that forced them to cease public-facing activities in March 2020. With the easing of restrictions in June 2020, riding lesson facilities were once again able to offer riding lessons and summer camps with modified protocols. Seventy-two riding facilities responded to an online survey to detail their operating protocols and the effect of these restrictions on their programs. Most facilities reported a decrease in the number of lessons they offered and the number of students per lesson. Less than half of the respondents offered summer camp programs. Both riding lessons and camp programs limited access to certain spaces in their barn or on their farm, and disinfected high contact areas and tack. Facemasks were not used by many facilities, however recent evidence suggests that facemasks should be worn even for outside activities. Despite the pandemic instigating challenges and hardships for many, there were nevertheless positive outcomes mentioned by many survey respondents. These included more time to spend with the horses, to attend to maintenance and repairs around the farm, more respect for barn rules and re-evaluation of business procedures and financial viability.

**Abstract:**

The COVID-19 pandemic has direct effects on the operations of riding lesson facilities and summer camps, with little government guidance on how to implement these. An online survey link was distributed to riding lesson facilities in Ontario. Descriptive statistics of respondents (*n* = 72) reported a decrease in both the number of riding lessons offered and the number of riding students per lesson. Scheduling riding times and limiting access to specific places on the farm ensured controlled access to the farms. Strict hygiene procedures were implemented including disinfecting high contact areas and shared tack. Summer camps followed the same procedures, although some farms chose not to offer camps at all. The use of facemasks was not prevalent in either riding lessons (43.3%) or camps (25%), likely because the activities took place outside. However, recent evidence shows that facemasks are perhaps even more important when outdoors, and it is recommended that riding lesson facilities re-evaluate their requirements for students and staff to wear facemasks while in the barn. In spite of the hardships, many positive aspects were noted including time to attend to repair and maintenance needs, scrutinizing business practices, more respect for barn rules, and more time to bond with the horses.

## 1. Introduction

On 17 March 2020, a state of emergency was declared in Ontario, Canada, resulting in the immediate shutdown of all nonessential businesses and services. This included horse boarding stables and riding lesson barns, although individuals required to maintain the minimum standards of care according to the National Farm Animal Care Council Code of Practice for the Care and Handling of Equines [1] were still allowed to come to work. However, public-facing programs such as riding lessons were required to cease. With this sudden loss of revenue, lesson barns in particular faced an economic crisis that directly affected the health and welfare of the horses. Over half of equine businesses in Canada surveyed in late March indicated they had less than one month of financial reserves to meet the expenses of feeding and caring for their horses, and only 19% of equine businesses were eligible for short-term income assistance from the federal government [2]. Equestrian Canada, the national governing body for equestrian activities in Canada, lobbied the federal government and were successful in including horse riding facilities in the Stage 1 Phase 2 of re-opening in mid-June (exact date varied by region). Equestrian facilities were included in “outdoor activities” that could resume operations with the implementation of modified health and safety requirements [3] including physical distancing and limited access to facilities. This meant extra cost for facilities to be able to implement these requirements.

Subsequent to the announcement made on 9 June 2020 by the Ontario provincial government that outdoor sports activities and summer day camps could resume operation [4], lesson barn owners were faced with a difficult decision: keep the farm gates closed to protect themselves from the threat of COVID-19, or reopen their business to try to regain revenue to feed themselves and their horses throughout the winter. No matter their situation, reopening during the pandemic has looked different for every barn owner. To gather insight on how various lesson barns have responded to this pandemic and the changing circumstances surrounding it, an online survey was used to gather qualitative data regarding riding lesson programs and day camps in Ontario.

## 2. Materials and Methods

A link to an online survey was sent by direct email to 157 lesson barns throughout Ontario representing various disciplines and having between 3–60+ school horses. The survey consisted of a total of 12 questions relating to the discipline of their barn, the precautions taken for lesson and camp programs, the effects of COVID-19 restrictions on their business and concerns barn owners had. The survey was available on the Qualtrics platform from 12–30 July 2020 and can be found in the Supplementary file S1. The raw data can be accessed here http://dx.doi.org/10.17632/rs5ym8b66g.1. Descriptive statistics on the qualitative data were determined in Microsoft Excel.

## 3. Results

Seventy-two farms located throughout Ontario, including two along the provincial border responded to the survey. The majority of farms taught hunter/jumper lessons (48%), 15% taught dressage, 11% taught eventing, and 7% taught western lessons. The remaining barns (19%) participated in other disciplines including saddleseat, trail riding, driving, horsemanship and therapeutic riding.

### 3.1. Lesson Programs

**Figure 1:**
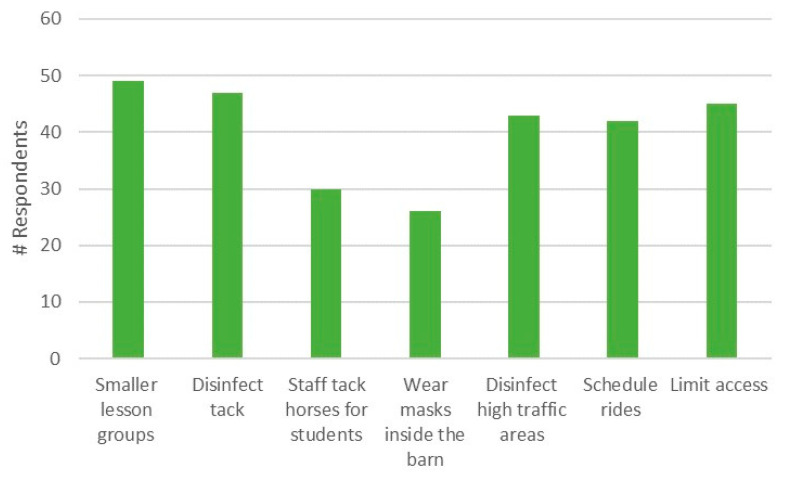
Precautions taken by survey respondents (*n* = 60) to ensure safety during COVID-19 restrictions for riding lessons at equestrian facilities in Ontario. Note that respondents could check all options that applied.

Of the 72 respondents, 60 (83%) ran a lesson program. The most widely reported COVID-19 safety protocols implemented included reducing the size of lesson groups, disinfecting tack and high-touch areas regularly, limiting access to certain areas of the barn, and scheduling rides so that there were never more than 10 people on the property (Figure 1).

Wearing of facemasks by staff and students while inside the barn was reported by 26 (43.3%) respondents. Eighteen respondents (25%) listed other COVID-19 safety procedures in place as well. These included:

- Riders must have their own detachable reins, saddle pad and grooming kit
- Tacking up outside only or using stalls for tacking up to keep the aisles clear and make it easier to follow social distancing measures; and
- Only teaching adults or those from the same household

For 67% of survey respondents, COVID-19 resulted in a decrease in the number of lessons they offer, either due to fewer students enrolling or due to the safety decisions of the barn owners that restricted numbers of students. Eighteen percent of facilities saw an increase in the number of lessons being taught, and 15% reported no change. On average, lessons ran with three students in a group, with a range of 1–12 students.

### 3.2. Summer Day Camps

Only 28 (39%) survey respondents indicated they were running day camps this summer. For those who continued to run day camps, 37.5% decreased the number of weeks they offered, 12.5% increased the number of weeks, and 50% reported no change.

The majority of camps ran with 10 or more children per week (62.5%) although numbers of campers ranged from four to eleven or more.

Many camps ran in alternative formats, with different activities and a change in structure to their regular program. Most facilities indicated they had to hire more staff to oversee smaller groups. Some notable responses of program structure changes included:

- Cancellation of overnight or lunch programs;
- Set cohorts of children staying with one counsellor the whole day and remaining in their small groups (no combined group games or activities);
- Fewer hours of camp per day and fewer days per week; and
- No new students could register for camps. Campers could only sign up for camp if they already rode at that barn.

**Figure 2:**
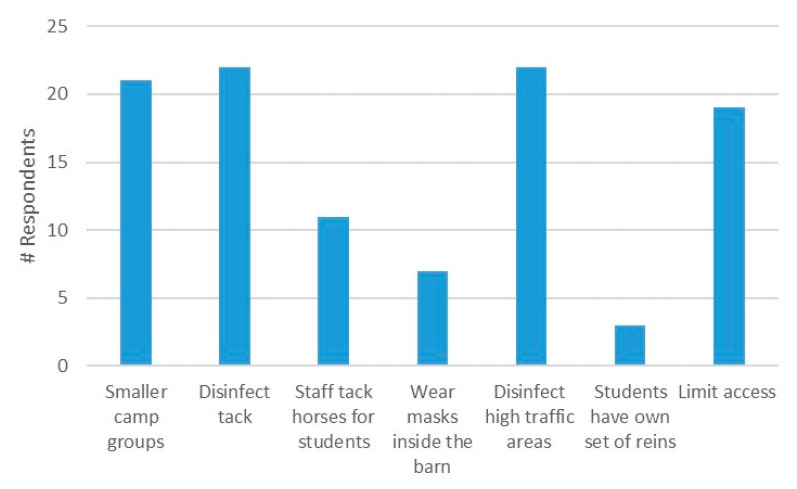
Precautions taken by survey respondents (*n* = 28) to ensure safety during COVID-19 restrictions for summer equestrian camps in Ontario. Note that respondents could check all options that applied.

The most common COVID-19 safety procedures taken to protect campers and staff were reported to be similar to those of regular lessons: disinfecting tack and high-touch areas, running day camps with smaller groups, and limiting access to certain areas of the barn (Figure 2). Many respondents also reported keeping temperature logs of all people at the facility and self-check-ins as precautions in accordance with the COVID-19 Guidance: Summer Day Camps (Version 2) [5] publication from the government of Ontario. Others reported campers being required to have their own grooming kits, lead ropes and saddle pads. Ensuring use of facemasks while inside the barn was only enforced by a small percentage (25%) of respondents.

Respondents noted their largest concern regarding running camps this summer was keeping everyone safe. Maintaining physical distancing, keeping everything disinfected and still providing fun activities for the campers were of equal concern, while not having enough campers register was of minor concern. Two facilities mentioned that they were worried about the possibility of having a camper test positive for COVID-19 and them being blacklisted as an outbreak source, even if the camper did not contract it from their facility.

### 3.3. Positive Aspects

In spite of the fact that the pandemic directly impacted revenue streams for riding lessons and summer camps, and many people have endured hardship as a result, survey respondents still referred to a number of positive aspects they have experienced during the lockdown and the phased-in return to business. Some positive responses referring to the period of lockdown included having a mental and physical break for self and horses, having time to attend to the many little things that one never has time for such as fixing fences, painting, maintenance and repair, and a closer evaluation of business practices to identify strengths and weaknesses and improve financial efficiencies. One respondent noted that it was the first time in years that they were able to spend Mother’s day and Father’s day with their family rather than being at a horse show. Farm owners also appreciated having the farm to themselves for some quiet time and were able to spend more time with their own horses.

Once riding lessons were allowed to resume, some of the positives noted included an increased interest in riding lessons, particularly from boys. Some farms assigned specific students to specific horses, unlike the usual practice of assigning different horses to students dependent on the week. Students appreciated not having to share their horse and reported a closer bond with their horse with more focus on training and less focus on showing. The switch to teaching more private and semi-private lessons rather than group lessons also allowed more one-on-one time with the instructor. Scheduling lessons became much easier as riding students were not constrained by typical 9:00 am-5:00 pm school or work hours; more daytime lessons could be offered. As students were clearly scheduled, it was also easy to know who was on the property at any time. Improved cleaning protocols were put in place and one respondent observed that their tack and equipment never looked so clean. There was a marked increase in awareness of barn rules and respect for following them. And finally, one respondent remarked that it was much less stressful to run a virtual parent show at the end of a camp session.

## 4. Discussion

Phase 2 of COVID-19-related restrictions allowed the resumption of riding activities in mid-June in Ontario. Our survey results showed that most riding schools and camps in Ontario resumed operations in a modified format, mainly by reducing the size of lesson groups, maintaining a strict hygiene protocol, and limiting access to the barns and property. However, some riding school and camp facilities chose to remain closed in the face of the pandemic. Regardless of the negative impacts of the pandemic, many survey respondents still voiced positive aspects that have resulted from the government-imposed restrictions.

Undoubtedly most riding lesson facilities are experiencing financial challenges due to loss of revenue. In Canada, horses are classified as livestock [2], nevertheless owners and riders tend to view them more as recreational or companion animals. This is reflected in the fact that while many farms earn at least one-third of their income from horse-related activities, the majority of them are not classified as agricultural, thus excluding them from agricultural-related government support programs in times of crises [2]. The majority of horse farms had less than one month reserve finances to care for their horses when revenue streams ceased [2]. The enforced downtime gave facility owners the opportunity to evaluate their business models and to make changes to improve financial efficiencies or make choices about the future of their business. Due to the loss of revenue, there was great financial pressure to resume operations immediately following the Ontario government’s green light for Phase 2 re-openings. Riding facilities quickly made the transition to revised operational protocols to be able to regain some of their income over the summer months. The Ontario Ministry of Health provided a guidance framework for summer day camps [5], but as riding facilities differ greatly from other typical children activities, riding facility owners were left to their own devices to determine appropriate operating procedures. The Ontario Ministry of Health recommendations for hygiene, physical distancing, and decreased group sizes needed to be adapted to farm situations. Riding facility owners were creative in addressing biosecurity concerns with adaptations such as requiring riders to provide their own grooming tools and reins to minimize contact with others. Riding facility owners also noted that riding school students and campers displayed an increased awareness of the importance of biosecurity and were more attentive to barn rules.

Disinfection of tack, equipment and high traffic areas was implemented in most riding facilities and riding camps. This is a good step in preventing the transmission of COVID-19 as studies have shown that while coronaviruses can live for up to nine days on non-porous surfaces, they can be almost immediately killed with antibacterial solutions [6]. Physical distancing is another important precaution to prevent the spread of COVID-19, however may be more difficult to implement in barns with narrow aisles or when assisting riding lesson students. The 2 m physical distancing rule was proposed based on studies done in the mid-19th century showing that most droplets produced by sneezing or coughing traveled less than 2 m distance, despite more recent evidence showing droplet spread up to 8 m away [7]. Most riding facilities approached physical distancing by limiting the number of people on the property or limiting access to certain areas of the facility to reduce the opportunities for close contact to other people. 

One aspect that appeared to be of least concern for both riding lessons and riding camps was the wearing of facemasks. The requirement for wearing facemasks in public was not implemented until early-to-mid July in Ontario [3], and much misinformation circulated about the wearing of facemasks. While the wearing of facemasks in public has become pervasive in many Asian countries, here in North America the wearing of a mask is still met with suspicion and viewed as an infringement of personal freedom [8]. Additionally, many myths originally surrounded the effectiveness of facemasks, with the belief that the mask may even increase the risk of infection when used incorrectly [9,10]. Wearing facemasks during physical activity may result in increased heart rate, increased respiration, shortness of breath, skin irritation, and challenges to thermoregulation [11,12]. Presumably the idea that riding generally takes place outdoors in a socially-distanced manner reduced the concern regarding the wearing of facemasks. However, recent research showed that the recommended 2 m physical distancing may not be far enough, as COVID-19 particles are lightweight and can carry as far as 11 m even in a very light 2 km/h wind [13]. This may encourage riders to consider wearing facemasks even while riding, even in an outdoor area where wind speed may increase the risk of airborne transmission of COVID-19 droplets. However, the advantage of outdoor settings allows the dilution of respiratory droplets much quicker than in indoor environments, which in turn decreases the risk of spread by almost 80% [14]. Regardless, repeated studies have shown the effectiveness of facemasks in reducing the spread of infection [8,15,16], and riding facilities should consider requiring all students and staff to wear facemasks at least when not mounted on a horse. However, knowledge on the spread of COVID-19 is still evolving, and physical distancing rules could be considered based on the factors of the activity undertaken and the ventilation level [7].

With the restrictions imposed by COVID-19, access and interaction with horses decreased suddenly, which may have downstream effects on animal health. For example, testing or vaccinating for diseases like Equine Infectious Anemia or Equine Influenza may be inaccessible due to unavailability of veterinarians or vaccines and reduced or diverted workloads at testing labs [17]. Foregoing regular health care such as vaccinations or hoof care may be particularly enticing for equestrian businesses where the pandemic has placed strains on their economic feasibility. Aside from the obvious animal welfare implications, animal health is an important driver of human health and should be observed even in difficult times. It was noted by survey respondents that the shutdown gave them more time to spend with their horses, to observe their overall health, and to give the horses a physical and mental break from their busy lesson schedules.

Access to horses plays an important role in the lives of those who interact with them. While horse owners may have been restricted to riding at specified times or only in outdoor locations, since the implementation of Phase 2, they were at least able to access their horse. However, riding lesson students remained more restricted due to limitations on the number of people allowed in a space such as a riding arena at any one time. Riding facility owners were creative in developing alternative approaches, and by assigning specific students to specific horses a partnership between horse and rider was enabled that hitherto fore was curbed by having to share a lesson horse with other lesson students. Riding students were able to concentrate more on creating a bond with their horse rather than pursuing horse show ribbons.

During the pandemic, physical activity has been highly recommended for the health benefits it provides, and as horseback riding is an outdoor activity, it was endorsed by most governments [18]. In response to the reduction in physical activity that the lockdown created, some riding lesson facilities saw an increase in lesson students once restrictions eased. In particular, more boys were participating in riding lessons, which is a positive step in a sport that is dominated by females [19]. Physical activity is directly linked to positive mental health, and decreased loneliness [20]. During a pandemic, acknowledging the importance of mental health is paramount as feelings of stress, anxiety, fear and sadness are heightened [21]. Here animals can play an important role on human mental health. Companion animals provide emotional support and prevent loneliness [22], and they become even more vital in times where social distancing and social isolation prevents regular interaction with other people. Indeed, pet ownership itself was considered more influential to combat loneliness than the strength of the human-animal bond [23]. Here equestrian facility owners may have an advantage as in spite of the anxiety that the pandemic is causing, they are still able to access their horses and perhaps even spend more time with them than previously.

## 5. Conclusions

The current pandemic has affected people in many ways, and in particular riding lesson facilities have been hard hit financially with continued costs for their horses together with reduced income from lessons. With the Phase 2 reopening in Ontario, riding lesson facilities and riding camps were allowed to resume activities if they chose. Alterations to their operating procedures included decreasing group sizes, limiting access to various spaces on the farm, and enforcing strict hygiene measures. Surprisingly, most facilities did not require the use of facemasks, and based on more recent evidence on the protective qualities of facemasks, their use is encouraged. Maintaining access to horses even through pandemic restrictions is important both for animal health and human mental health. 

## Supplementary Materials

The raw data is available online at https://www.mdpi.com/2076-2615/10/12/2412/s1. S1: Survey questions.
