# Supplementary material for: The Effect of the COVID-19 Pandemic on Riding Lesson Barns and Summer Camps in Ontario
Source: Animals (Basel). 2020 Dec 17;10(12):2412. doi: 10.3390/ani10122412 (PMC7766263; doi:10.3390/ani10122412)
Supplement: Supplementary file 1 [file animals-10-02412-s001.pdf]

**COVID-19 Effects on Equestrian Lesson and Camp Programs**

Q1 - What is the main discipline of your barn? (check all that apply)

| | |
|---|---|
| ☐ | Hunter/Jumper |
| ☐ | Dressage |
| ☐ | Eventing |
| ☐ | Western |
| ☐ | Saddle Seat |
| ☐ | Other (please describe) |

Q2 - Where are you located?

Q3 - Does your facility run a lesson program?

| | |
|---|---|
| ☐ | Yes |
| ☐ | No |

Q4 - What precautions are you taking to ensure everyone's safety when they come to your barn for lessons? (check all that apply)

| | |
|---|---|
| ☐ | Smaller lesson groups (indicate max number of students) |
| ☐ | Disinfecting tack |
| ☐ | Having staff tack horses for students |
| ☐ | Ensuring staff and students are wearing masks inside the barn |
| ☐ | Disinfecting high traffic areas and objects (i.e. light switches, doorknobs etc.) |
| ☐ | Scheduling rides so there is never more than 10 people on property |
| ☐ | Limiting access to certain areas of the barn |
| ☐ | Other (please describe) |

Q5 - How has COVID-19 affected the number of lessons you are running?

| | |
|---|---|
| ☐ | An increase in the number of lessons |
| ☐ | A decrease the number of lessons |
| ☐ | No change |

Q6 - Is your facility planning on running camps this summer?

| | |
|---|---|
| ☐ | Yes |
| ☐ | No |

Q7 - How has COVID-19 affected the number of weeks of camp you are running?

| | |
|---|---|
| ☐ | An increase in the number of camp weeks |
| ☐ | A decrease the number of camp weeks |
| ☐ | No change |

Q8 - What is the maximum number of children you would allow to attend your camp on a weekly basis?

Q9 - Is the structure of your camps relatively the same as past years? How is it the same? How is it different? What does the average day of your camp look like?

Q10 - What precautions are you taking to ensure everyone's safety when they come to your barn for camp? (check all that apply)

| | |
|---|---|
| ☐ | Smaller camper groups (indicate max number of students) |
| ☐ | Disinfecting tack |
| ☐ | Having staff tack horses for students |
| ☐ | Ensuring staff and students are wearing masks inside the barn |
| ☐ | Disinfecting high traffic areas and objects (i.e. light switches, doorknobs etc.) |
| ☐ | Students have their own set of reins for the week |
| ☐ | Limiting access to certain areas of the barn |
| ☐ | Other<br>(please describe) |

Q11 - What are your biggest concerns regarding running camps this summer? (rank from highest to lowest)

| | |
|---|---|
| 1 | Not having enough kids sign up to run camps |
| 2 | Keeping everyone safe and limiting the risk of COVID-19 transmission |
| 3 | Ensuring physical distancing |
| 4 | Keeping everything clean and disinfected |
| 5 | Providing activities that are safe and still enjoyable |
| 6 | Other |

Q12 - Describe any positive aspects of the COVID-19 pandemic you have experienced. This can be either horse-related or not horse-related.
